# Supplementary material for: RAS/PI3K pathway mutations sensitise epithelial ovarian cancer cells to a PARP/NAMPT inhibitor combination
Source: Commun Biol. 2025 Dec 19;9:6. doi: 10.1038/s42003-025-09223-0 (PMC12764903; doi:10.1038/s42003-025-09223-0)
Supplement: Supplementary file 2 — Supplementary Information [file 42003_2025_9223_MOESM2_ESM.pdf]

SUPPLEMENTARY FIGURES

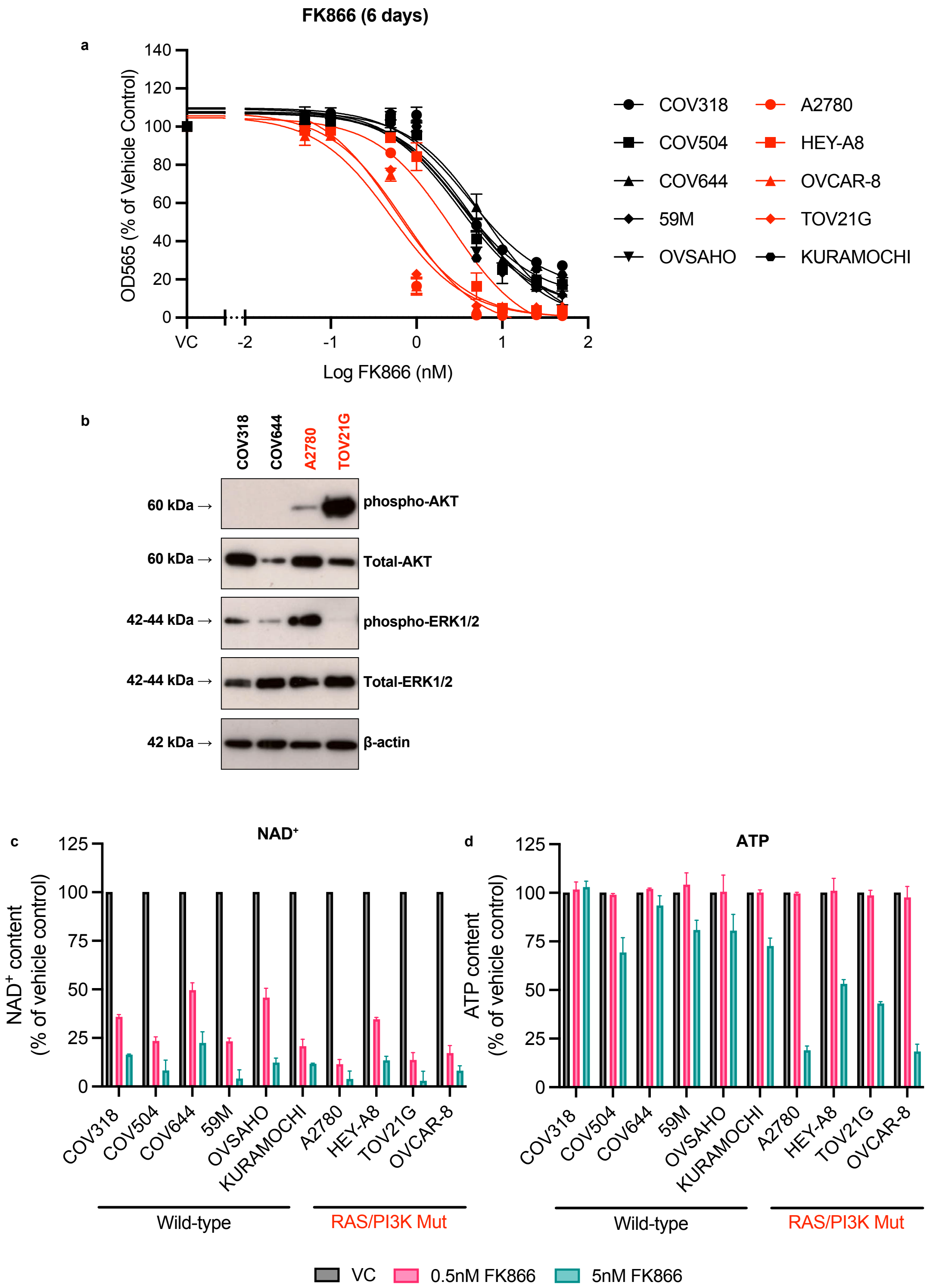

Supplementary Figure. 1: FK866 treatment depletes NAD$^+$ and ATP pools to a greater extent in RAS/PI3K-mutant EOC cell lines. a) EOC cell lines FK866 sensitivity after 6-days treatment. Cell biomass was measured using the SRB assay. **b)** AKT and ERK1/2 signalling in RAS/PI3K-wildtype (COV318 and COV644) and -mutant (A2780 and TOV21G) cell lines. EOC cell lines were treated with vehicle (0.1% DMSO) or FK866 (0.5nM or 5nM) for **c)** 24-hours to measure NAD$^+$ content using the NAD/NADH-Glo^{TM} assay or for **d)** 48-hours to measure ATP content using the CellTiter-Glo$^{®}$ 2.0 assay. **c-d)** Data was normalised to cell biomass (SRB assay). Data is the average ± SD of three independent experiments.

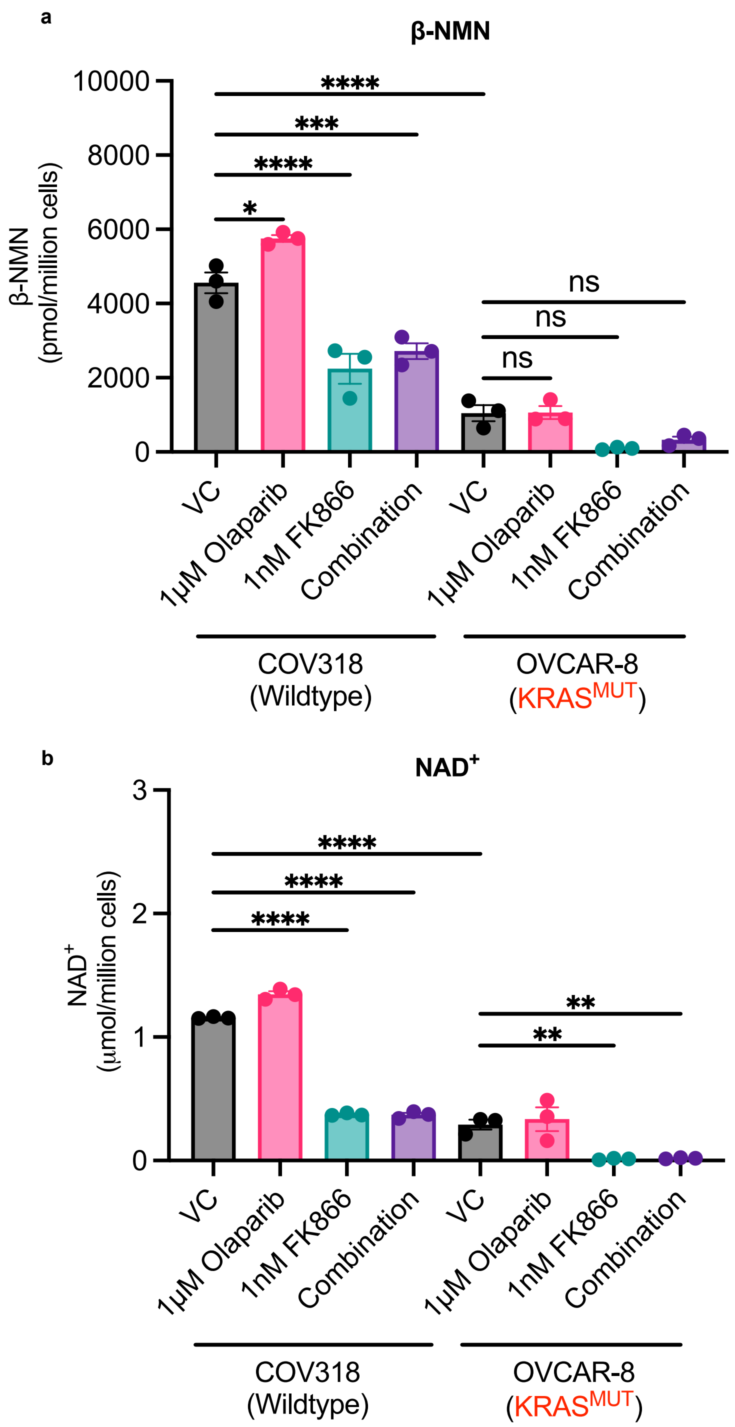

Supplementary Figure. 2: Combined olaparib and FK866 treatment depletes β-NMN and NAD$^+$ pools to a greater extent in RAS-mutant OVCAR-8 cells. a-b) COV318 and OVCAR-8 cells were treated for 24-hours with vehicle, olaparib, FK866 or the combination, and then samples were extracted for UPLC-MS/MS analysis. The concentration of **a)** β-NMN and **b)** NAD$^+$ were calculated using a standard curve. Data is the average ± SD of three independent experiments. Statistical significance was determined using 2-way ANOVA followed by Turkey's multiple comparisons test (p < 0.05, **p < 0.01, *** p < 0.001, **** p < 0.0001).

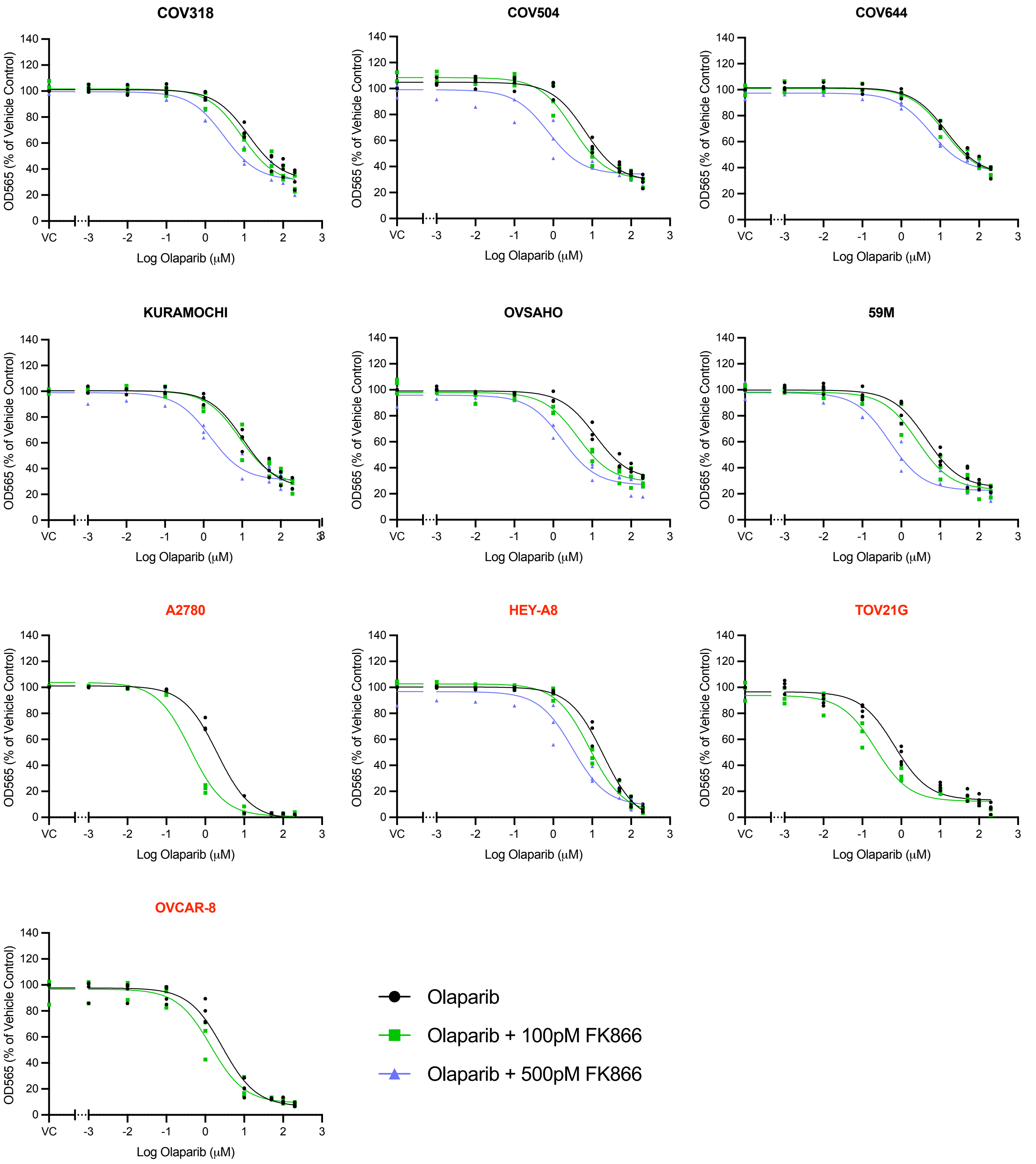

Supplementary Figure. 3: FK866 potentiates the cytotoxic effects of olaparib in EOC cell lines in 2D culture. EOC cell lines were co-treated with olaparib and indicated doses of FK866 for 6-days. Cell biomass was measured using the SRB assay. Data is from three independent experiments.

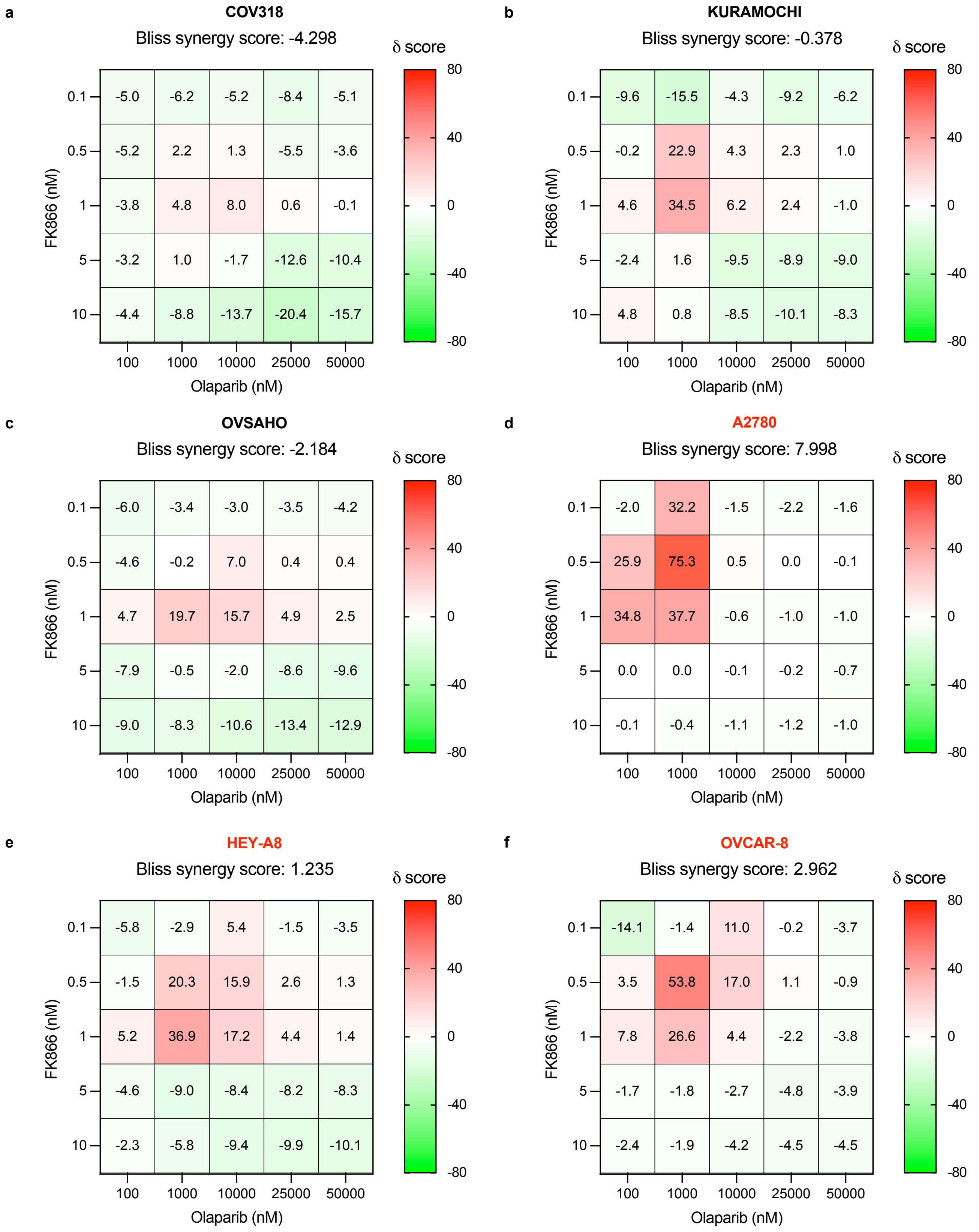

Supplementary Figure. 4: Synergy scores for the olaparib and FK866 in EOC cell lines in 2D culture. EOC cell lines were co-treated with olaparib and indicated doses of FK866 for 6-days. Bliss synergy maps of the combination are shown for each cell line. Higher δ scores (red) represent increasing synergy, whereas decreasing scores (green) demonstrate increasing antagonism. Cell biomass was measured using the SRB assay. Data is the average ± SD of three independent experiments. Synergy scores were calculated using SynergyFinder[60].

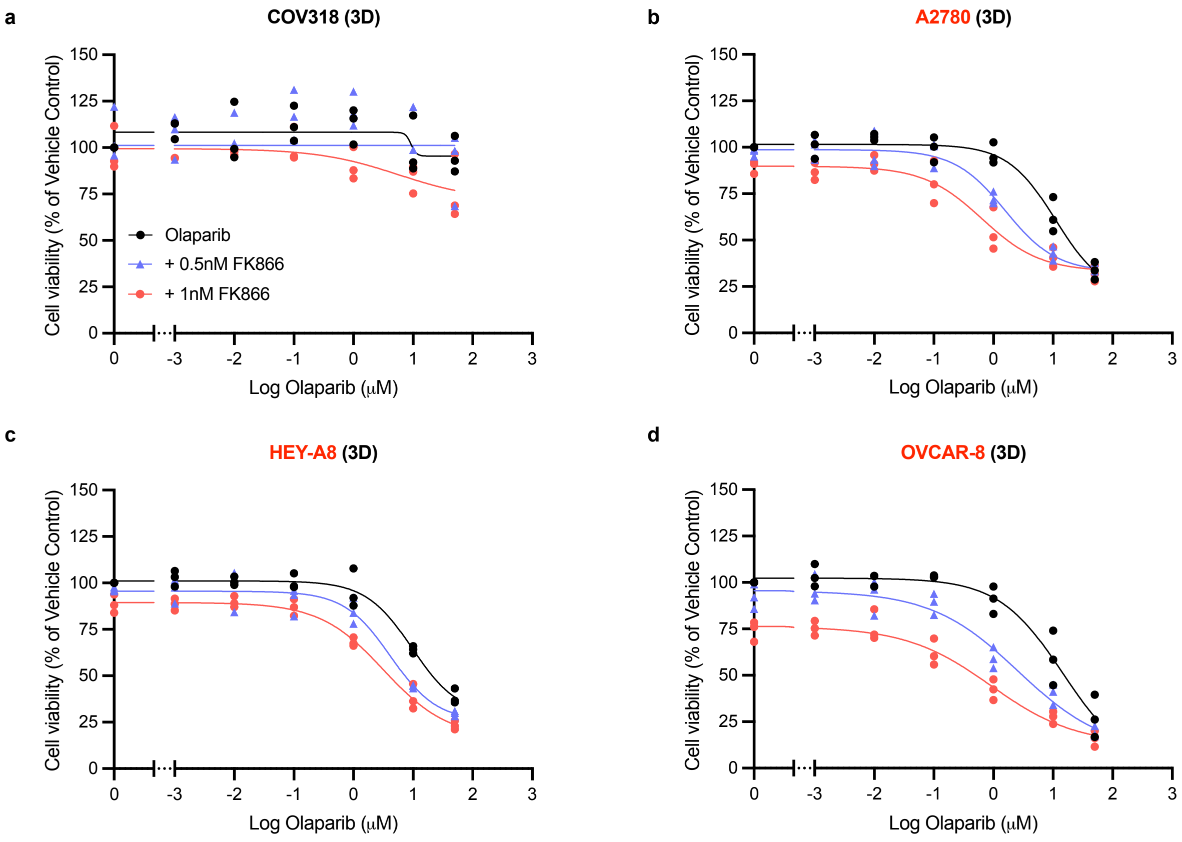

Supplementary Figure. 5: FK866 potentiates the cytotoxic effects of olaparib in RAS/PI3K-mutant EOC cell lines in 3D culture. a) COV318, **b)** A2780, **c)** HEY-A8 and **d)** OVCAR-8 spheroids were co-treated with olaparib and FK866 (0.5nM or 1nM) to assess their sensitivity to the combination after 6-days treatment. Cell viability was measured using the CellTitre-Glo® 3D assay. Data are from three independent experiments.

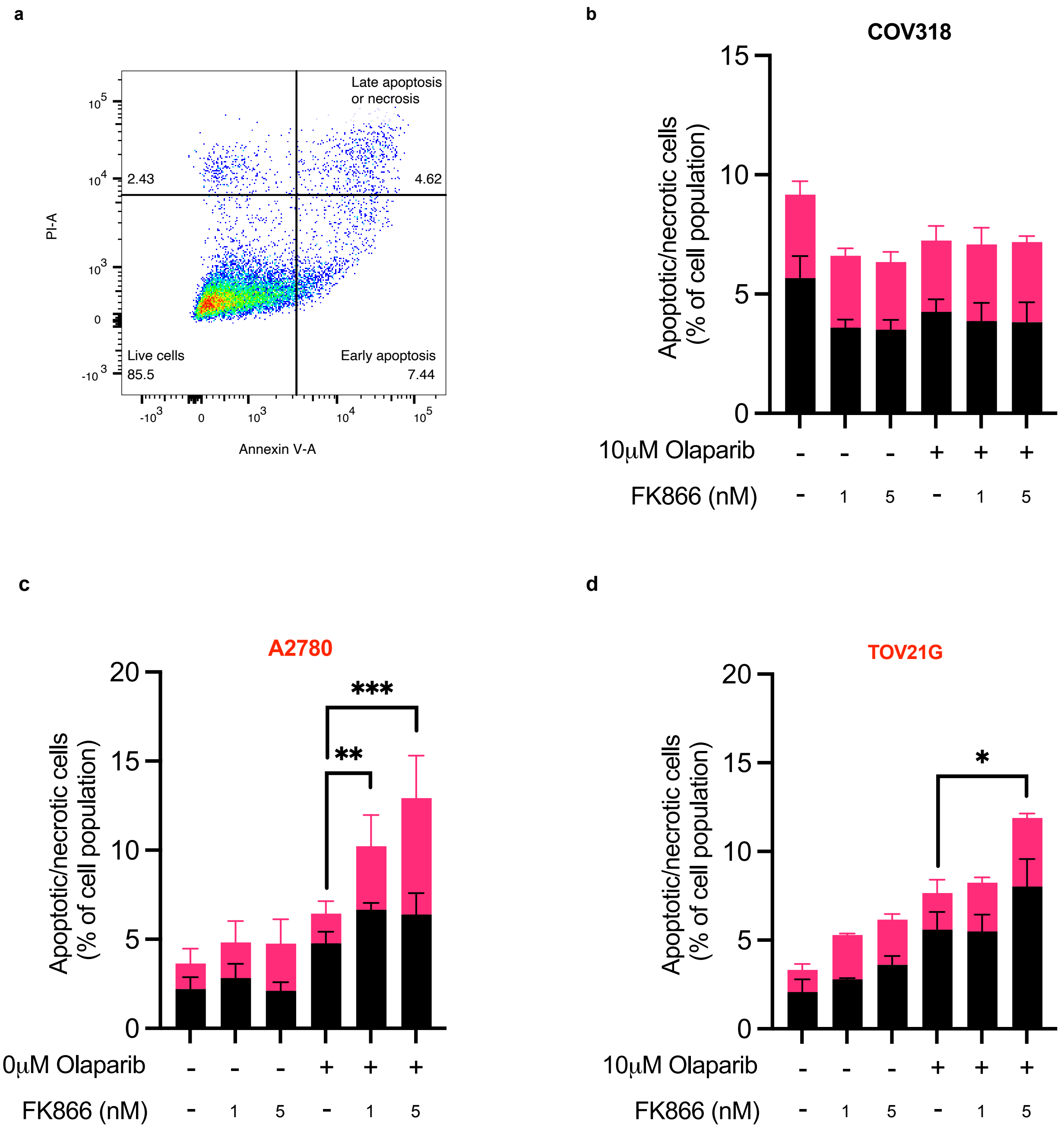

Supplementary Figure. 6: The combination induces apoptosis/necrosis in RAS/PI3K mutant EOC cell lines. a) Example of gating used for the Annexin V apoptosis assay. **b)** COV318, **c)** A2780 and **d)** TOV21G cells were treated for 24-hours with vehicle, olaparib, FK866 or the combination before measuring the level of apoptosis/necrosis. Data is the average ± SD of three independent experiments. Statistical significance was determined using 2-way ANOVA followed by Turkey's multiple comparisons test ($p < 0.05$, **$p < 0.01$, *** $p < 0.001$, **** $p < 0.0001$).

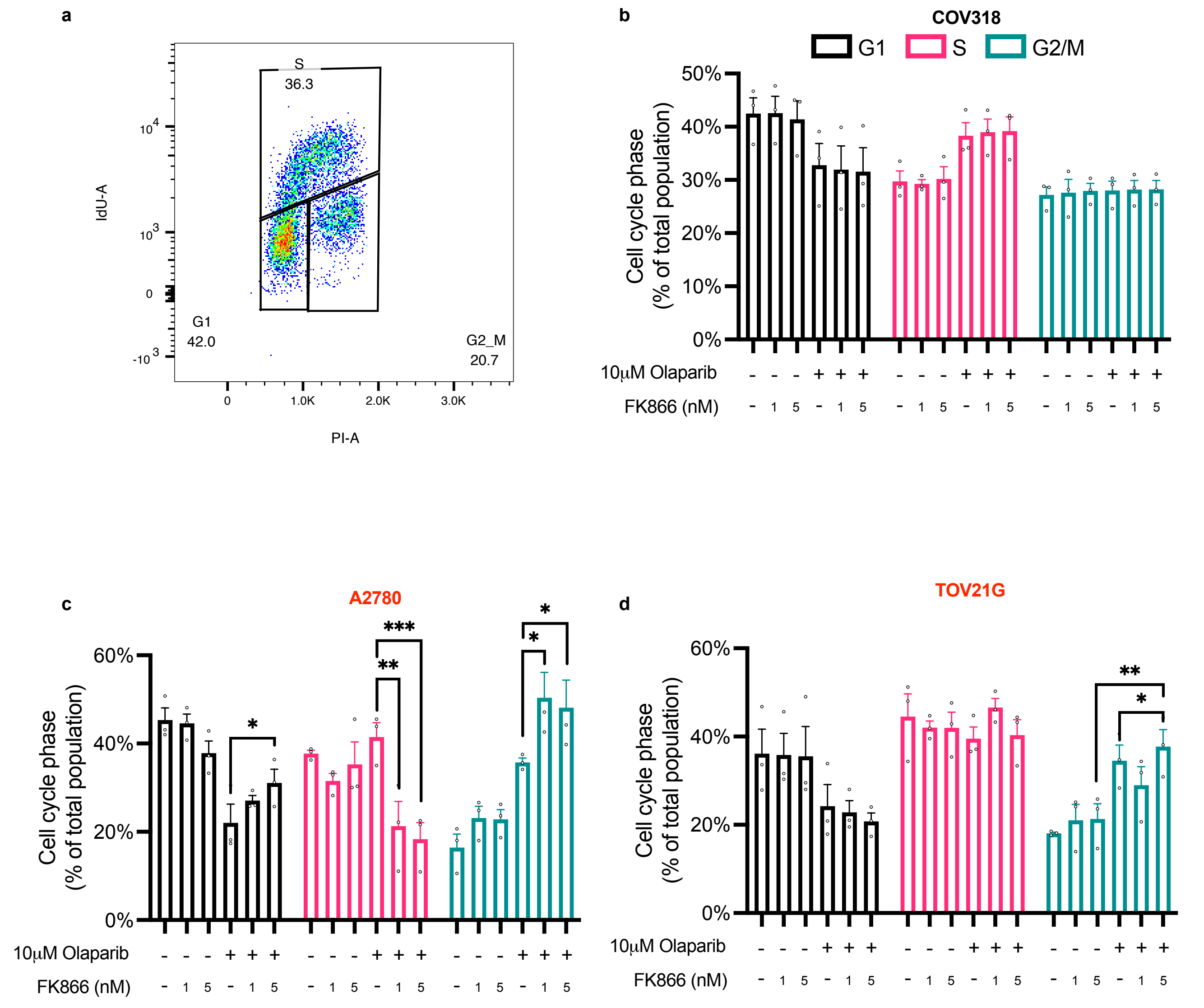

Supplementary Figure. 7: The combination upregulates G2/M arrest in RAS/PI3K-mutant EOC cell lines. a) Example gating for the determination of cells in G1, S or G2/M phases by flow cytometry. **b)** COV318, **c)** A2780 and **d)** TOV21G cells were treated for 24-hours with vehicle, olaparib, FK866 or the combination before measuring cell cycle progression. Data is the average ± SD of three independent experiments. Statistical significance was determined using 2-way ANOVA followed by Turkey's multiple comparisons test ($p < 0.05$, **$p < 0.01$, *** $p < 0.001$, **** $p < 0.0001$).

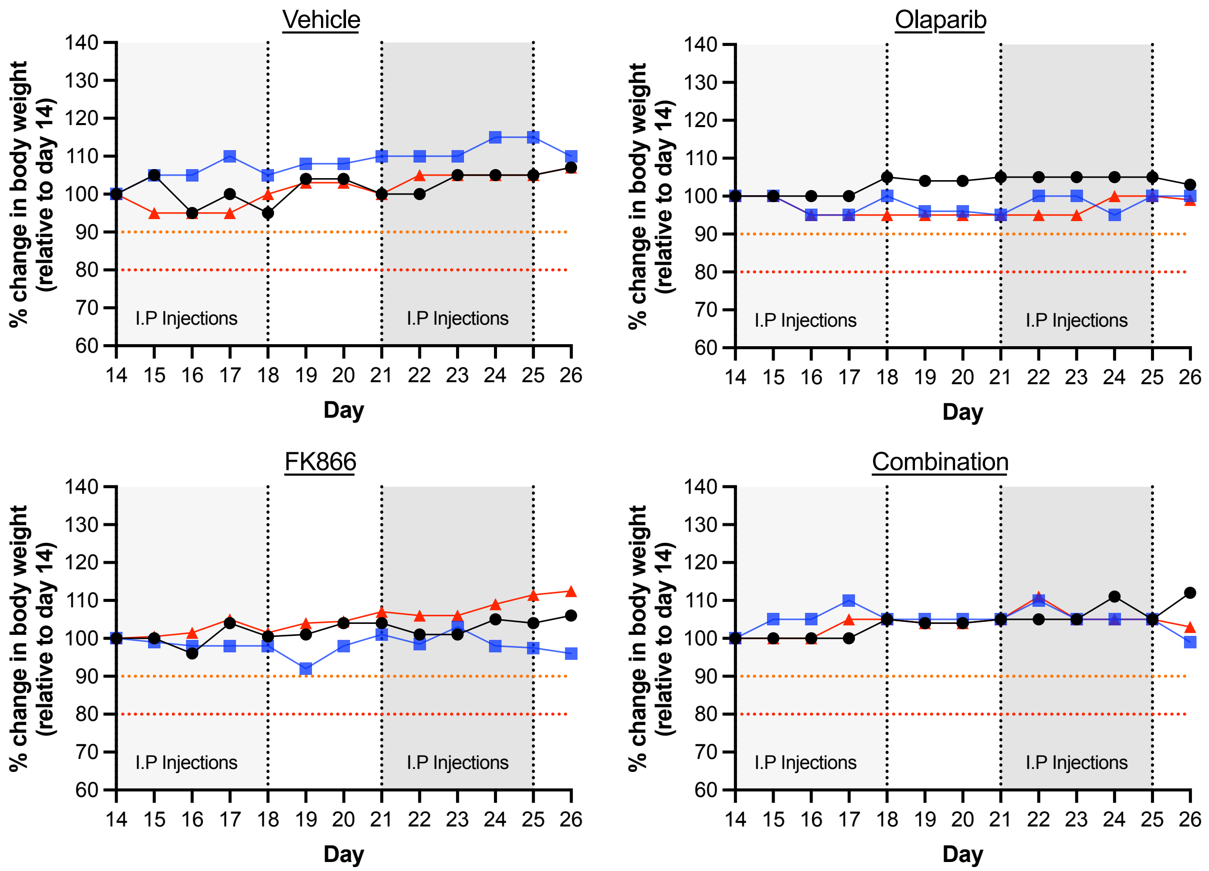

Supplementary Figure. 8: Relative body weight changes of C57BL/6J mice during the *in vivo* endpoint experiment. Relative body weight changes were measured each morning. In each condition data is from three separate mice. Orange and red dotted lines on the y-axis indicate the threshold for 10% and 20% (maximum limit) drops in body weight, respectively.

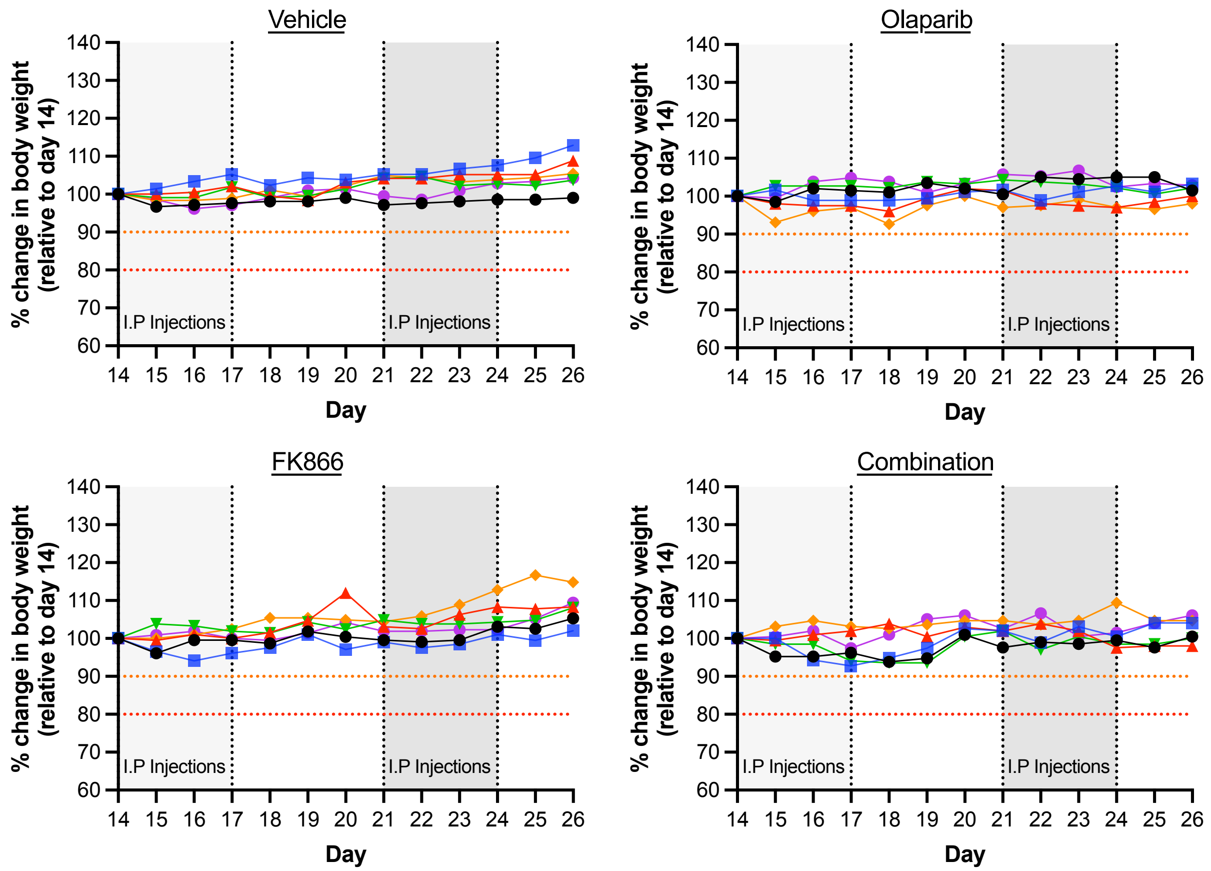

Supplementary Figure. 9: Relative body weight changes of C57BL/6J mice during the *in vivo* survival experiment. Relative body weight changes were measured each morning. In each condition data is from six separate mice. Orange and red dotted lines on the y-axis indicate the threshold for 10% and 20% (maximum limit) drops in body weight, respectively.

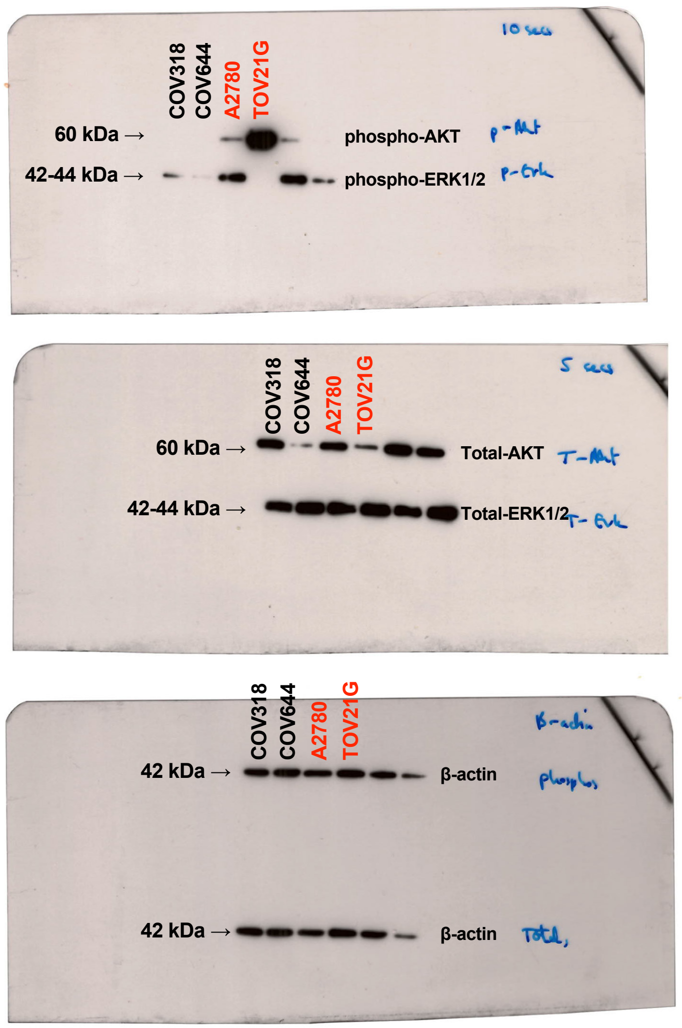

Supplementary Figure. 10: Uncropped blot images. Uncropped blot images from supplementary figure 1B. Scans of the phosphor-AKT and phosphor-ERK1/2, total-AKT and total-ERK1/2 and the Beta-actin blots are shown. COV318, COV644, A2780 and TOV21G cells are included on blots from left to right.

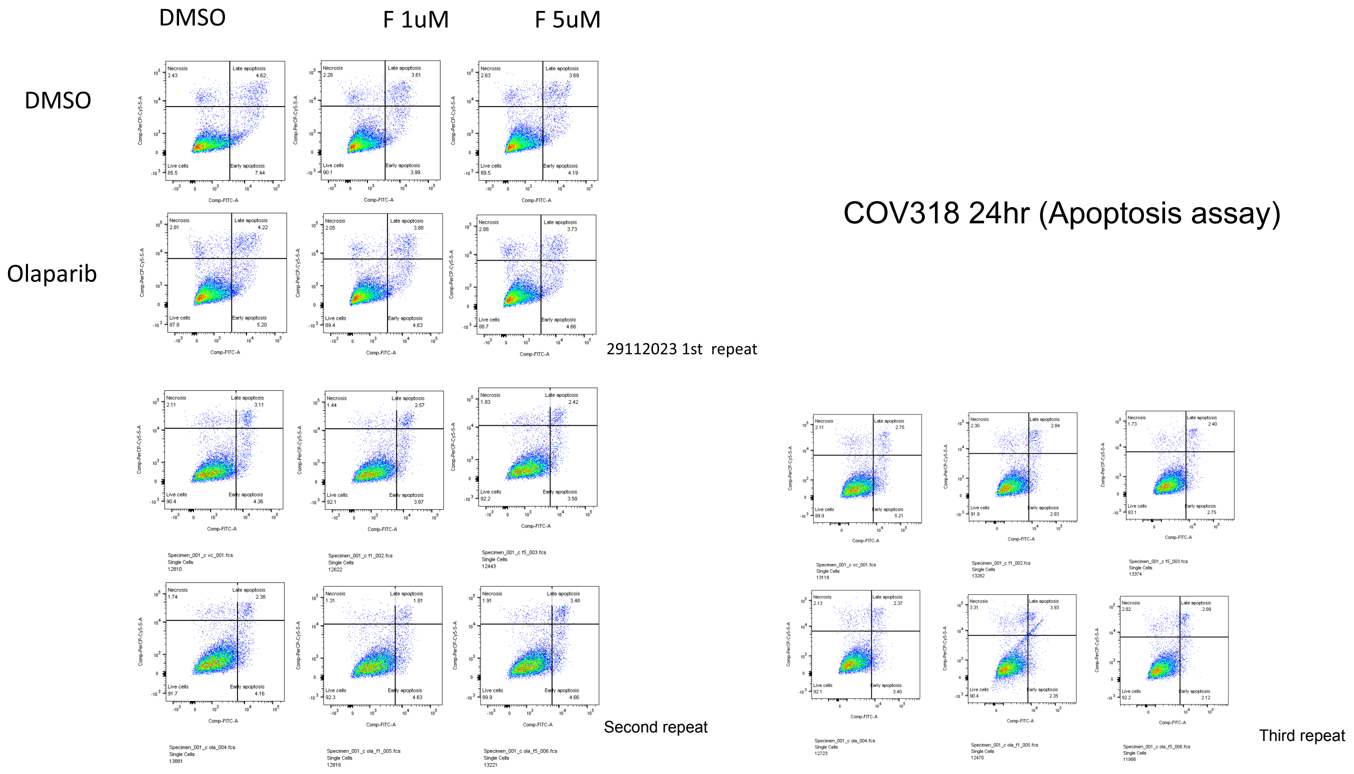

Supplementary Figure. 11: Annexin V apoptosis/necrosis assay in the COV318 cell line. FACS gating strategy used for the Annexin V apoptosis assay. Individual replicates from experiments using the COV318 cell line are shown. Cells were treated for 24-hours with vehicle, olaparib, FK866 or the combination before measuring the level of apoptosis/necrosis.

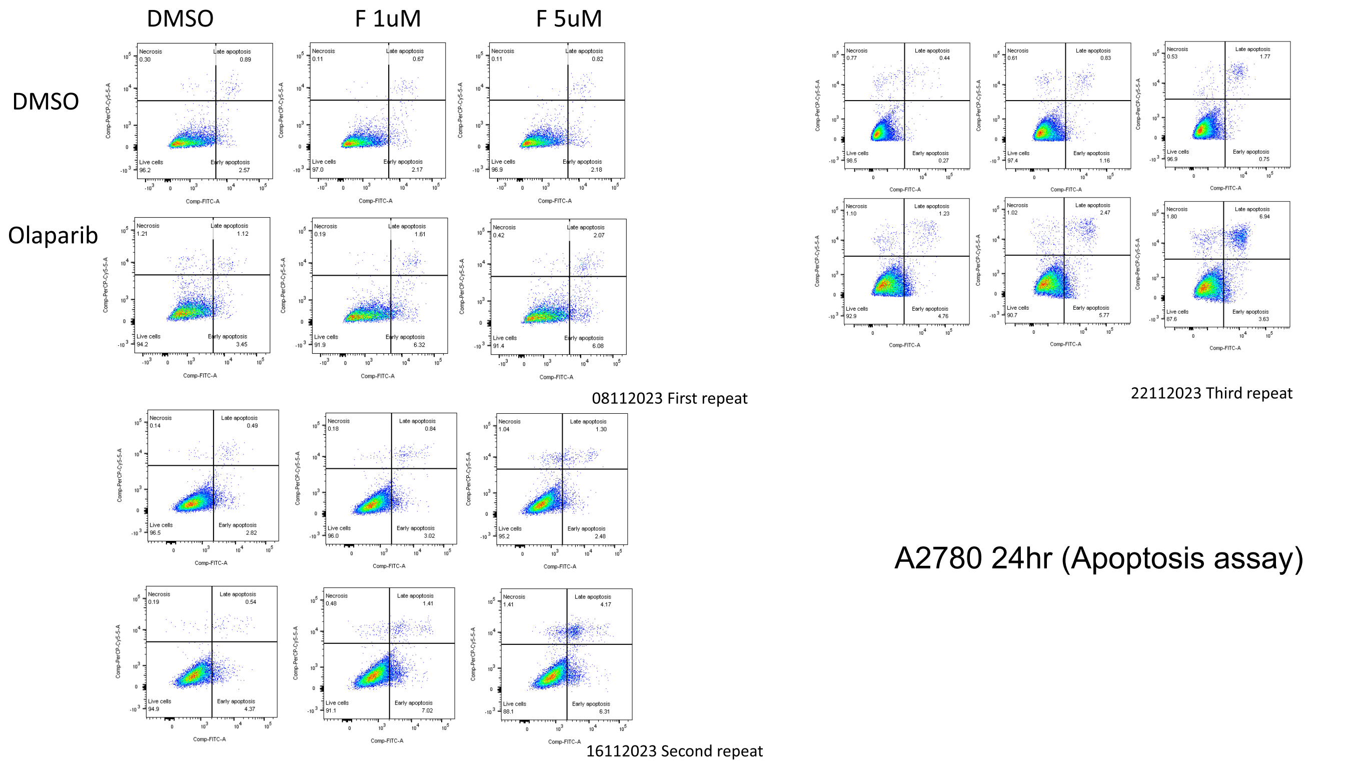

Supplementary Figure. 12: Annexin V apoptosis/necrosis assay in the A2780 cell line. FACS gating strategy used for the Annexin V apoptosis assay. Individual replicates from experiments using the A2780 cell line are shown. Cells were treated for 24-hours with vehicle, olaparib, FK866 or the combination before measuring the level of apoptosis/necrosis.

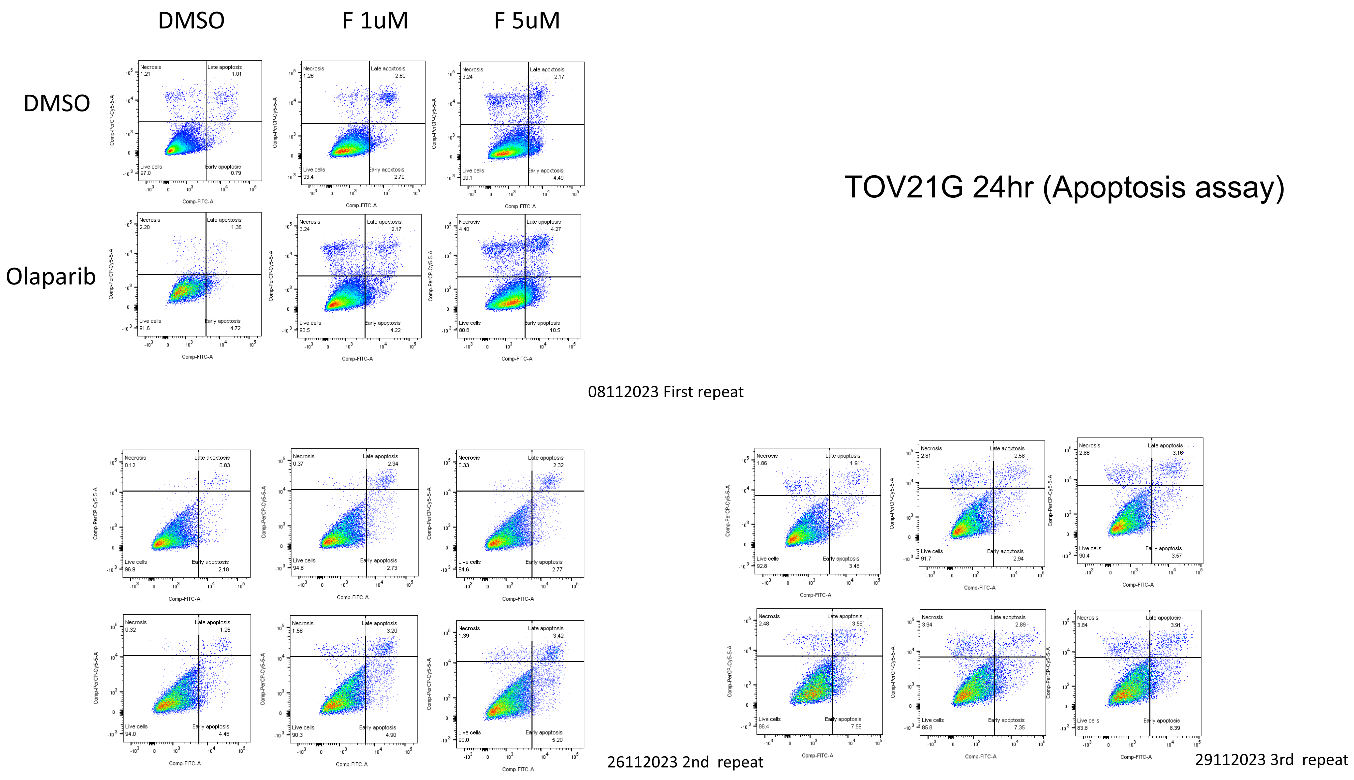

Supplementary Figure. 13: Annexin V apoptosis/necrosis assay in the TOV21G cell line. FACS gating strategy used for the Annexin V apoptosis assay. Individual replicates from experiments using the A2780 cell line are shown. Cells were treated for 24-hours with vehicle, olaparib, FK866 or the combination before measuring the level of apoptosis/necrosis.

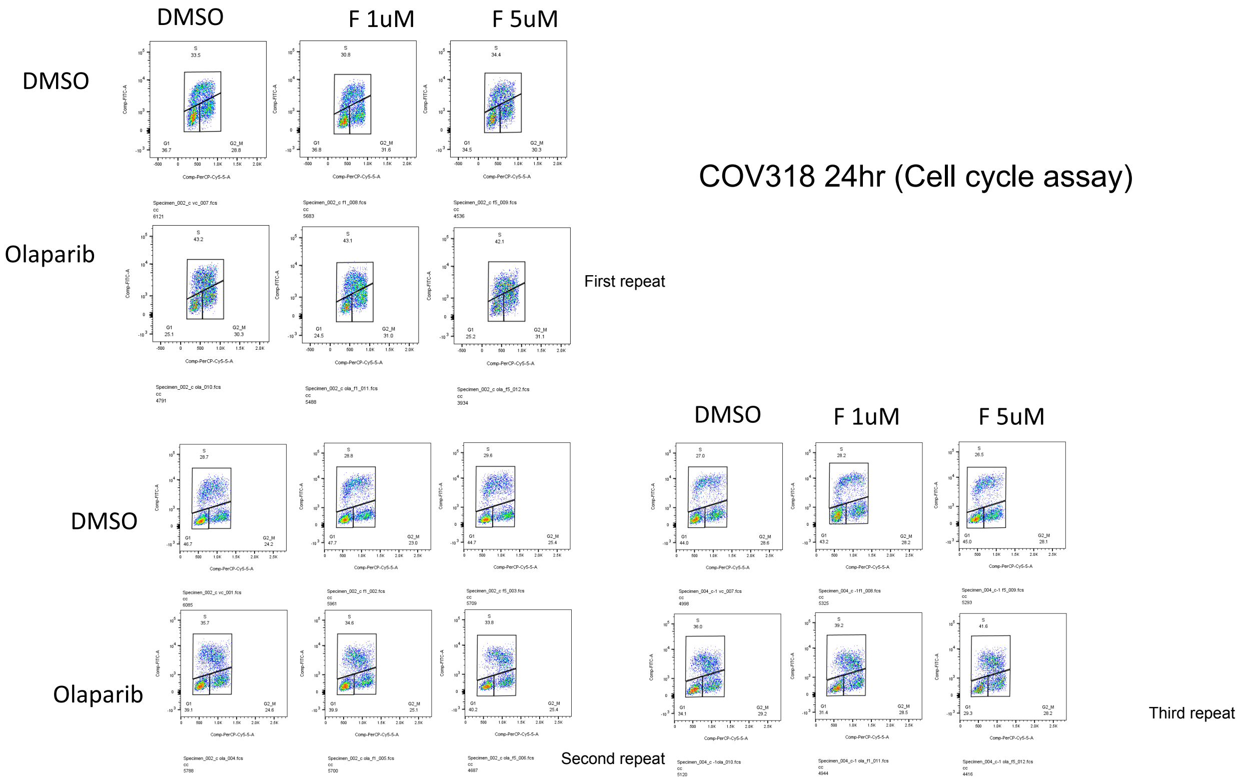

Supplementary Figure. 14: Cell cycle assay in the COV318 cell. FACS gating strategy used for the Annexin V apoptosis assay. Individual replicates from experiments using the COV318 cell line are shown. Cells were treated for 24-hours with vehicle, olaparib, FK866 or the combination before measuring the number of cells in G1, S or G2/M phases by flow cytometry.

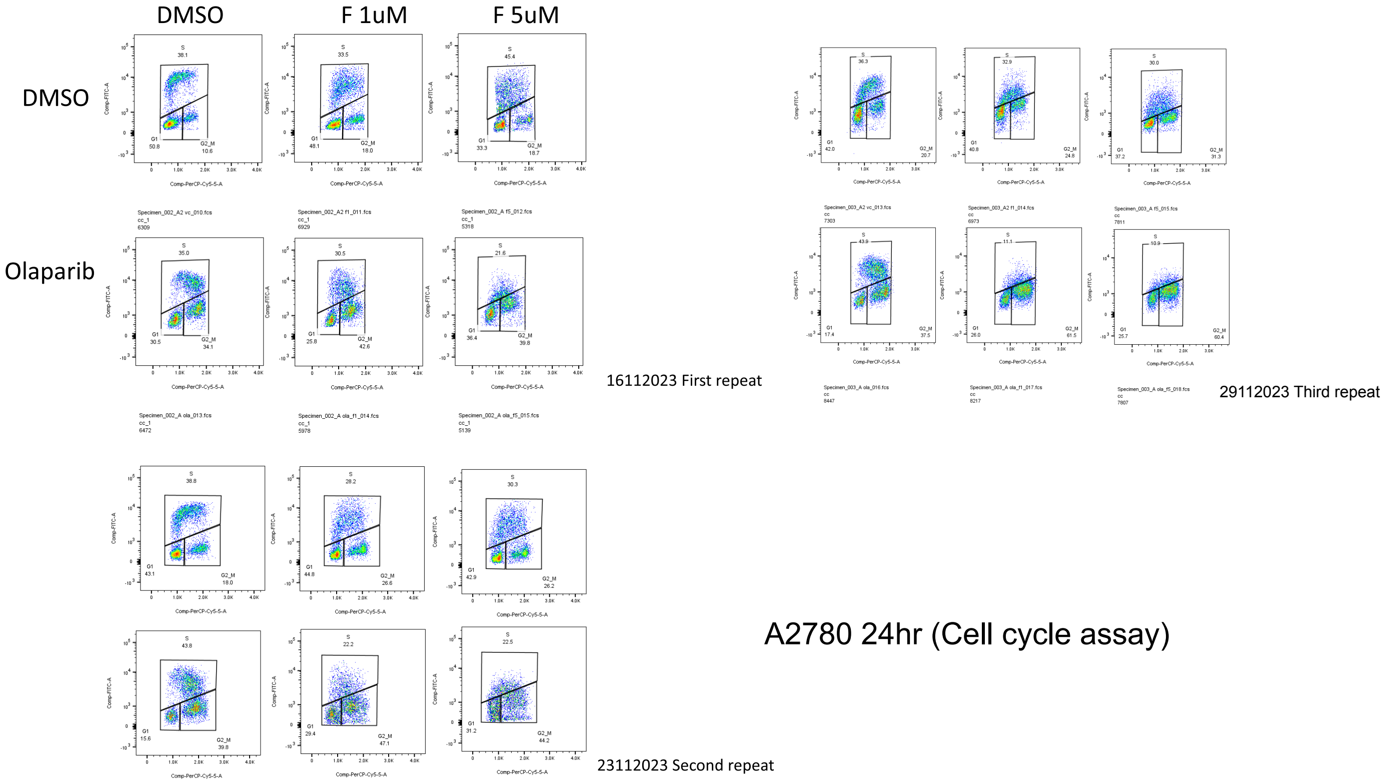

Supplementary Figure. 15: Cell cycle assay in the A2780 cell. FACS gating strategy used for the Annexin V apoptosis assay. Individual replicates from experiments using the A2780 cell line are shown. Cells were treated for 24-hours with vehicle, olaparib, FK866 or the combination before measuring the number of cells in G1, S or G2/M phases by flow cytometry.

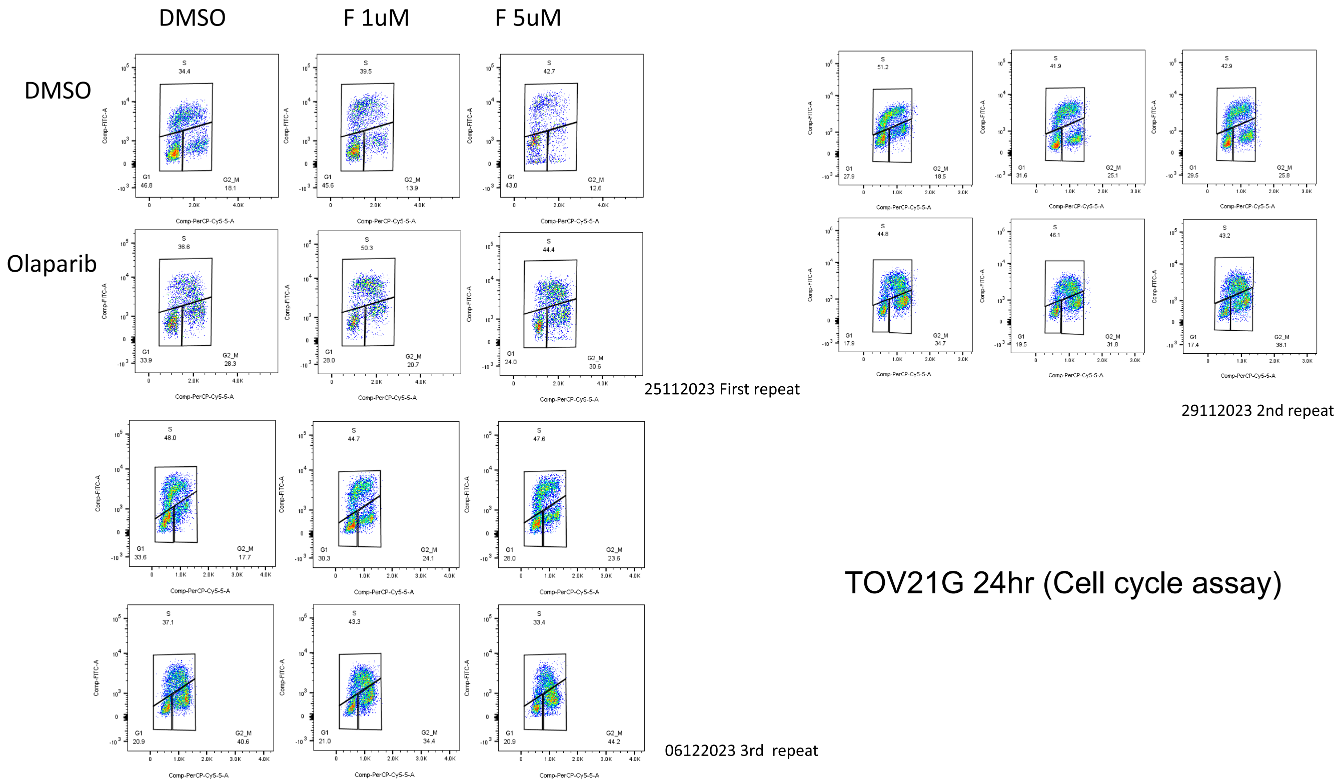

Supplementary Figure. 16: Cell cycle assay in the TOV21G cell. FACS gating strategy used for the Annexin V apoptosis assay. Individual replicates from experiments using the TOV21G cell line are shown. Cells were treated for 24-hours with vehicle, olaparib, FK866 or the combination before measuring the number of cells in G1, S or G2/M phases by flow cytometry.

SUPPLEMENTARY TABLES

Supplementary Table. 1: RP UPLC gradient elution in positive mode.

Time (min)	Flow rate (µL/min)	Mobile Phase A (%)	Mobile Phase B (%)
0.00	600	99.5	0.5
2.00	600	99.5	0.5
5.00	600	85.0	15.0
10.00	600	0.5	99.5
13.00	600	0.5	99.5
13.10	600	99.5	0.5
15.00	600	99.5	0.5

Supplementary Table. 2: MRM parameters for the RP UPLC-MS/MS NMN/NAD$^+$ assay

Metabolite	Q1	Q3	RT (min)	DP	CE
NAD_1	664.0	136.0	0.88	70	70
NMN_1	335.0	123.0	0.68	100	20

Only main parent/daughter ions used in experiment are shown.

Supplementary Table. 3: HILIC UPLC gradient elution in in positive mode.

Time (min)	Flow rate (µL/min)	Mobile Phase A (%)	Mobile Phase B (%)
0.00	500	95.0	5.0
1.00	500	95.0	5.0
8.00	500	50.0	50.0
9.00	500	50.0	50.0
9.10	500	95.0	5.0
15.00	500	95.0	5.0

Supplementary Table. 4: MRM parameters for the HILIC-positive UPLC-MS/MS NAD$^+$ assay

Metabolite	Q1	Q3	RT (min)	DP	CE
NAD1	664	136	20	70	70

Only the main parent/daughter ion used in the experiment is shown.
