# Supplementary material for: RAS/PI3K pathway mutations sensitise epithelial ovarian cancer cells to a PARP/NAMPT inhibitor combination
Source: Commun Biol. 2025 Dec 19;9:6. doi: 10.1038/s42003-025-09223-0 (PMC12764903; doi:10.1038/s42003-025-09223-0)
Supplement: Supplementary file 3 — Description of Additional Supplementary Files [file 42003_2025_9223_MOESM3_ESM.pdf]

Description of Additional Supplementary Files

File name: Supplementary Data 1
Description: Excel file with the data used for each figure panel. Each datasheet is labelled to highlight which figure panel data corresponds to.
